# Supplementary material for: Single-cell Analysis Reveals Inter- and Intratumour Heterogeneity in Metastatic Breast Cancer
Source: J Mammary Gland Biol Neoplasia. 2023 Dec 8;28(1):26. doi: 10.1007/s10911-023-09551-z (PMC10709262; doi:10.1007/s10911-023-09551-z)
Supplement: Supplementary file 3 — Additional file 3: Extended Fig. 1. a, Table summarizing the numbers of cells sequenced and processed for analysis for each model / site of origin after quality control. Extended Fig. 2. a, Clustering and distribution of cells from different cell cycle stages and library complexity (PDG) before correction (top row) and after correction (lower row) for each model. b, Clustering performed over tSNE produces 16 cell clusters (c) that have unbalanced representation of cells coming from different models. Extended Fig. 3. a, Cell clusters identified separately from each individual model, show overlapping enrichment in Hallmark genets. b, Corrected clusters generated from all the cells irrespectively of the model of origin, showing the cluster composition according to site of origin. c, tSNE plot showing clustering of the corrected clusters. Extended Fig. 4. a, tSNE plot showing the cell repartition across the 3 superclusters defined via GSEA. b, Barplot showing the supercluster composition according to site of origin. c, Kaplan-Meyer plot showing the RFS in patients with basal-like BC (n= 442) according to expression of the upregulated transcripts defining supercluster A. d, Kaplan-Meyer plot showing the RFS in patients with basal-like BC (n= 442) according to expression of the upregulated transcripts defining supercluster B. e, Kaplan-Meyer plot showing the RFS in patients with basal-like BC (n= 442) according to expression of the upregulated transcripts defining supercluster C. [file 10911_2023_9551_MOESM3_ESM.pdf]

a

Model	Tumour	Lung Metastases
PDX4	151	17
PDX3	23	115
PDX2	177	114
PDX1	298	314
MDA-MB-231	221	93
Total	870	653

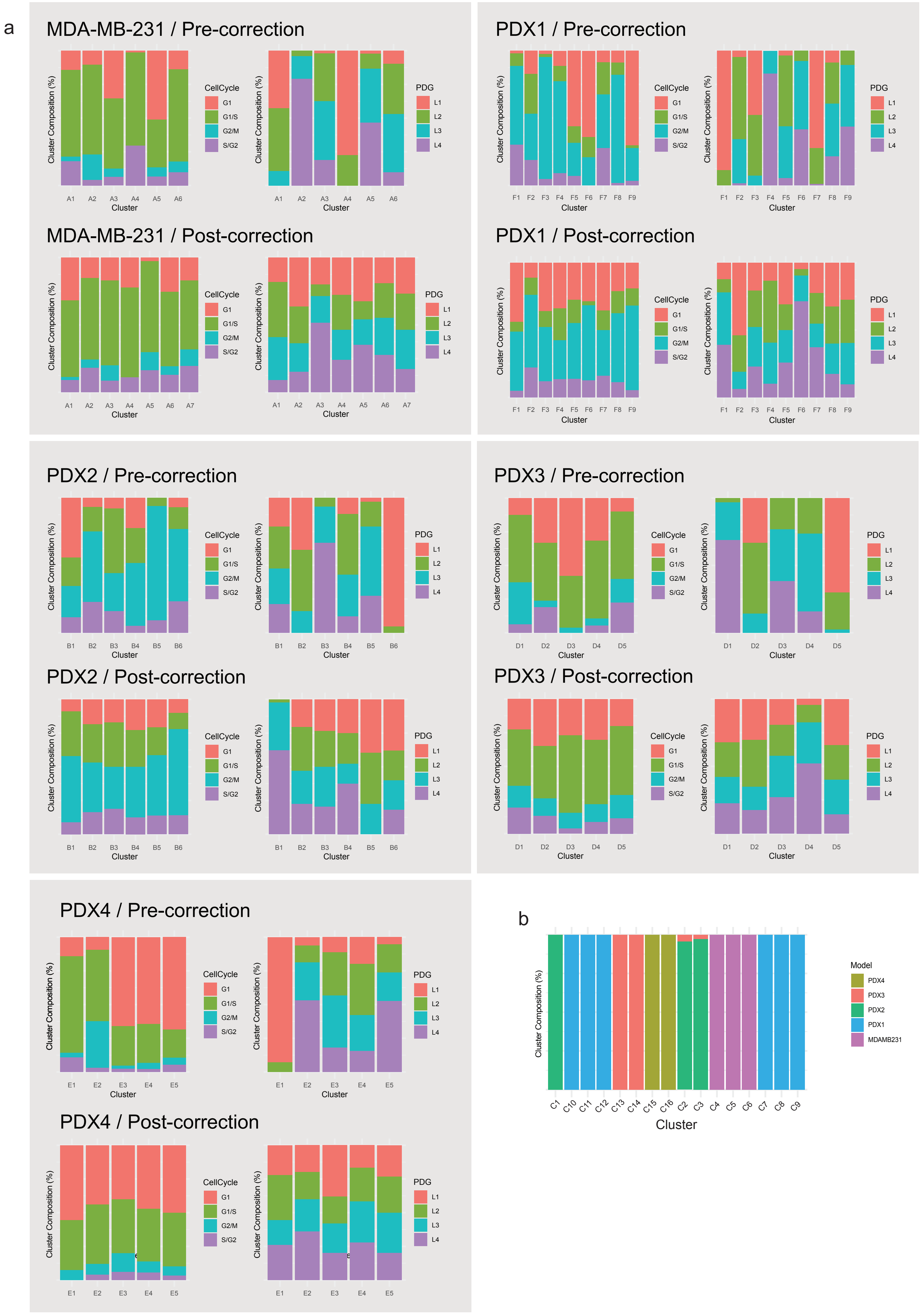
a
MDA-MB-231 / Pre-correction
Cluster Composition (%)
A1 A2 A3 A4 A5 A6
Cluster
CellCycle
G1
G1/S
G2/M
S/G2
PDG
L1
L2
L3
L4
MDA-MB-231 / Post-correction
A1 A2 A3 A4 A5 A6 A7
PDX1 / Pre-correction
F1 F2 F3 F4 F5 F6 F7 F8 F9
PDX1 / Post-correction
PDX2 / Pre-correction
B1 B2 B3 B4 B5 B6
PDX2 / Post-correction
PDX3 / Pre-correction
D1 D2 D3 D4 D5
PDX3 / Post-correction
PDX4 / Pre-correction
E1 E2 E3 E4 E5
PDX4 / Post-correction
b
Cluster Composition (%)
C1 C10 C11 C12 C13 C14 C15 C16 C2 C3 C4 C5 C6 C7 C8 C9
Cluster
Model
PDX4
PDX3
PDX2
PDX1
MDAMB231

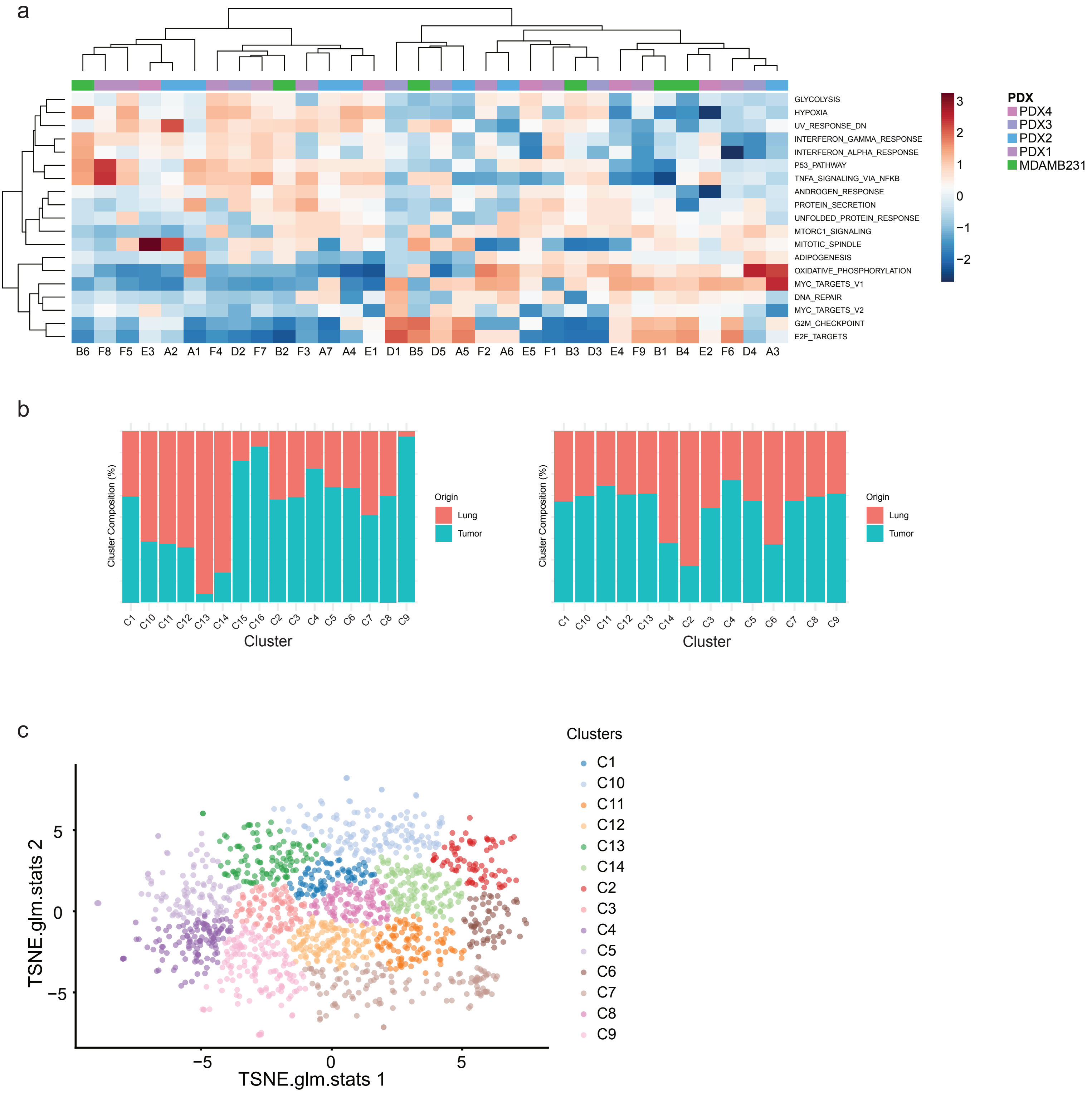
a
GLYCOLYSIS
HYPOXIA
UV_RESPONSE_DN
INTERFERON_GAMMA_RESPONSE
INTERFERON_ALPHA_RESPONSE
P53_PATHWAY
TNFA_SIGNALING_VIA_NFKB
ANDROGEN_RESPONSE
PROTEIN_SECRETION
UNFOLDED_PROTEIN_RESPONSE
MTORC1_SIGNALING
MITOTIC_SPINDLE
ADIPOGENESIS
OXIDATIVE_PHOSPHORYLATION
MYC_TARGETS_V1
DNA_REPAIR
MYC_TARGETS_V2
G2M_CHECKPOINT
E2F_TARGETS
B6 F8 F5 E3 A2 A1 F4 D2 F7 B2 F3 A7 A4 E1 D1 B5 D5 A5 F2 A6 E5 F1 B3 D3 E4 F9 B1 B4 E2 F6 D4 A3
3
2
1
0
−1
−2
PDX
PDX4
PDX3
PDX2
PDX1
MDAMB231
b
Cluster Composition (%)
C1 C10 C11 C12 C13 C14 C15 C16 C2 C3 C4 C5 C6 C7 C8 C9
Cluster
Origin
Lung
Tumor
Cluster Composition (%)
C1 C10 C11 C12 C13 C14 C2 C3 C4 C5 C6 C7 C8 C9
Cluster
Origin
Lung
Tumor
c
Clusters
C1
C10
C11
C12
C13
C14
C2
C3
C4
C5
C6
C7
C8
C9
TSNE.glm.stats 2
5
0
−5
−5
0
5
TSNE.glm.stats 1

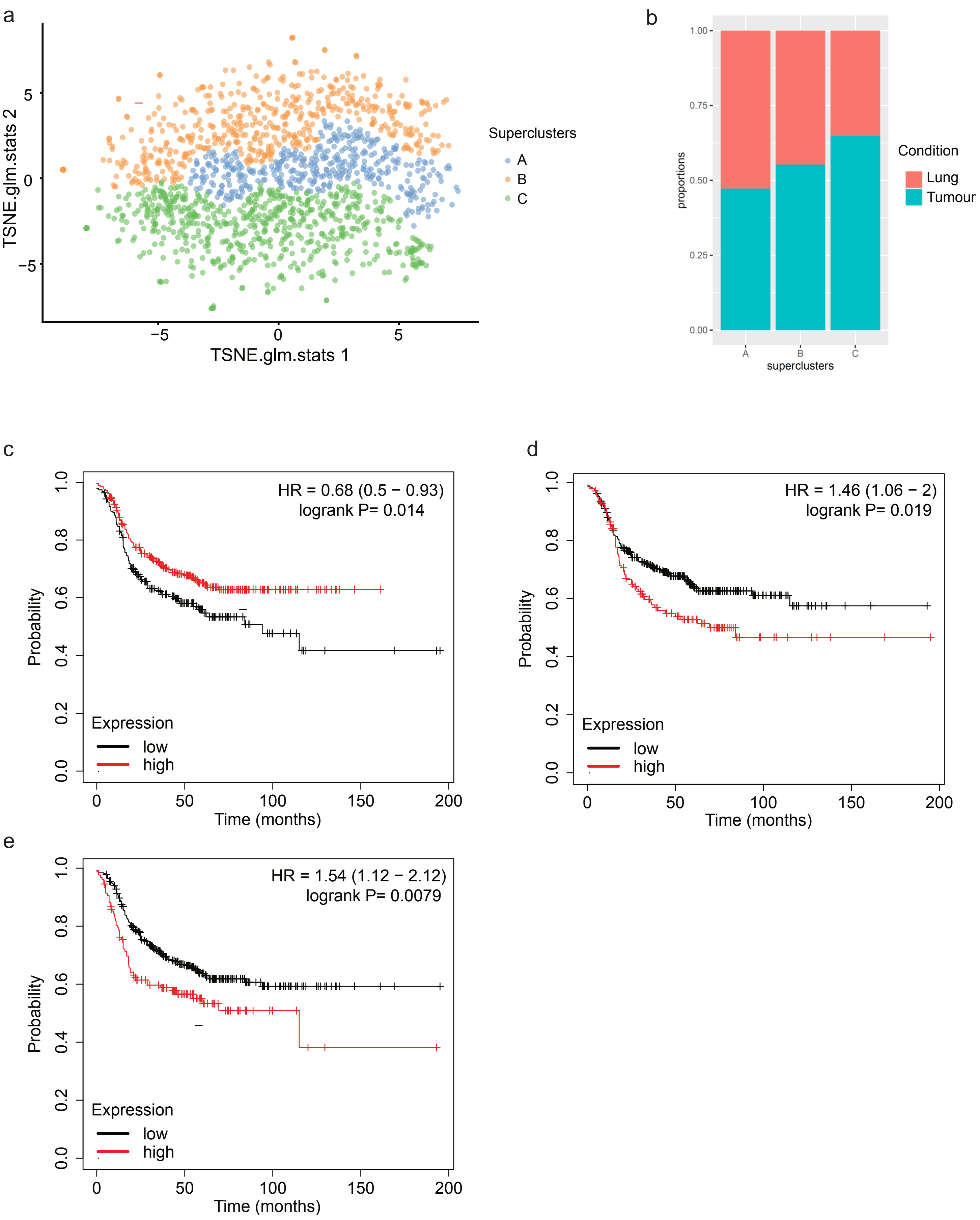
a
TSNE.glm.stats 2
TSNE.glm.stats 1
5
0
−5
−5
0
5
Superclusters
A
B
C
b
proportions
1.00
0.75
0.50
0.25
0.00
A
B
C
superclusters
Condition
Lung
Tumour
c
HR = 0.68 (0.5 − 0.93)
logrank P= 0.014
Probability
Time (months)
Expression
low
high
d
HR = 1.46 (1.06 − 2)
logrank P= 0.019
Probability
Time (months)
Expression
low
high
e
HR = 1.54 (1.12 − 2.12)
logrank P= 0.0079
Probability
Time (months)
Expression
low
high
0.0
0.2
0.4
0.6
0.8
1.0
0
50
100
150
200

Extended Figure 1

a, Table summarizing the numbers of cells sequenced and processed for analysis for each model / site of origin after quality control

Extended Figure 2

a, Clustering and distribution of cells from different cell cycle stages and library complexity (PDG) before correction (top row) and after correction (lower row) for each model.

b, Clustering performed over tSNE produces 16 cell clusters (c) that have unbalanced representation of cells coming from different models.

Extended Figure 3

a, Cell clusters identified separately from each individual model, show overlapping enrichment in Hallmark genets

b, Corrected clusters generated from all the cells irrespectively of the model of origin, showing the cluster composition according to site of origin

c, tSNE plot showing clustering of the corrected clusters

Extended Figure 4

a, tSNE plot showing the cell repartition across the 3 superclusters defined via GSEA

b, Barplot showing the supercluster composition according to site of origin

c, Kaplan-Meyer plot showing the RFS in patients with basal-like BC (n=442) according to expression of the upregulated transcripts defining supercluster A

d, Kaplan-Meyer plot showing the RFS in patients with basal-like BC (n=442) according to expression of the upregulated transcripts defining supercluster B

e, Kaplan-Meyer plot showing the RFS in patients with basal-like BC (n=442) according to expression of the upregulated transcripts defining supercluster C
